# Case Report: A case of autoinflammatory disease with a novel *NLRP12* variant—clinical presentation and successful treatment with baricitinib

**DOI:** 10.3389/fmed.2026.1723843

**Published:** 2026-02-06

**Authors:** Wenjing Wang, Qing Li, Xin Ma, Liqing Zhou, Dongfeng Ge, Hua Fan, Hongwei Jiang, Xiaofei Shi

**Affiliations:** 1 Department of Rheumatology and Immunology, The First Affiliated Hospital, College of Clinical Medicine, Henan University of Science and Technology, Luoyang, China; 2 Department of Cell Biology and Genetics, Guangxi Medical University, Nanning, China; 3 Department of Pathology, The First Affiliated Hospital, College of Clinical Medicine, Henan University of Science and Technology, Luoyang, China; 4 Office of Research & Innovation, The First Affiliated Hospital, College of Clinical Medicine, Henan University of Science and Technology, Luoyang, China; 5 Henan Key Laboratory of Rare Diseases, Endocrinology and Metabolism Center, The First Affiliated Hospital, College of Clinical Medicine, Henan University of Science and Technology, Luoyang, China

**Keywords:** autoinflammatory disease, baricitinib, case report, interleukin-6, JAK inhibitor, *NLRP12*

## Abstract

*NLRP12*-associated autoinflammatory disease (*NLRP12*-AID) is a rare monogenic disorder. The p.Glu619Gln (c.1855G > C) variant in *NLRP12* is classified as a variant of uncertain significance (VUS), with no previously reported clinical cases. We describe a 16-year-old Chinese girl with a 9-year history of periodic high fevers (>39 °C), cold-triggered urticarial rashes, and polyarticular arthralgia. Previous misdiagnoses included recurrent infections and juvenile idiopathic arthritis. Laboratory tests showed elevated levels of interleukin-6 (IL-6) and acute-phase reactants. A lymph node biopsy confirmed necrotizing lymphadenitis. Extensive testing ruled out infections, autoimmune diseases, and cancers. Whole-exome sequencing identified a heterozygous *NLRP12* p.Glu619Gln variant. Structural analysis with AlphaFold2 predicted that the mutation causes local structural destabilization and impairs function. After treatment with baricitinib (2 mg/day), the patient experienced rapid symptom relief within 2 weeks, with IL-6 levels decreasing from 17.8 pg./mL to 2.1 pg./mL and maintained clinical control over 12 months of follow-up. This is the first reported case providing multiple lines of evidence linking the *NLRP12* p.Glu619Gln VUS to a typical autoinflammatory profile. Characteristic symptoms, inflammatory markers, histopathology, and a notable response to JAK inhibition support the diagnosis. Our findings suggest reclassifying this variant as likely pathogenic and propose baricitinib as a targeted therapy for *NLRP12*-AID.

## Highlights

- This is the first clinical case report of the *NLRP12* p.Glu619Gln (c.1855G > C) variant.
- The patient exhibited a typical *NLRP12*-AID phenotype, including cold-triggered urticaria and recurrent fevers.
- Objective evidence of autoinflammation included elevated IL-6, acute-phase reactants, and biopsy-confirmed necrotizing lymphadenitis.
- The patient exhibited a quick and sustained complete response to the JAK inhibitor baricitinib.
- This case offers important clinical evidence to reclassify this VUS as likely pathogenic.

## 1 Introduction

*NLRP12*-associated autoinflammatory disease (*NLRP12*-AID), also known as familial cold autoinflammatory syndrome 2 (FCAS2), is a rare autosomal dominant disorder caused by mutations in the *NLRP12* gene (1). The disease typically appears in childhood with recurring fevers, cold-triggered urticarial rashes, joint pain, and elevated acute-phase reactants (2). The *NLRP12* p.Glu619Gln (c.1855G > C) variant, located in the conserved HD2 domain, is predicted by computational analysis to be damaging but is still classified as a variant of uncertain significance (VUS) in ClinVar (3), with the protein sequence referenced from UniProt (P59046) (4). To date, there have been no clinical cases reported (5, 6). Here, we present the first clinical case of a patient with a classic autoinflammatory phenotype carrying this novel *NLRP12* variant, including details of the clinical presentation, diagnostic process, and successful treatment with baricitinib.

## 2 Case description

**Figure 1:**
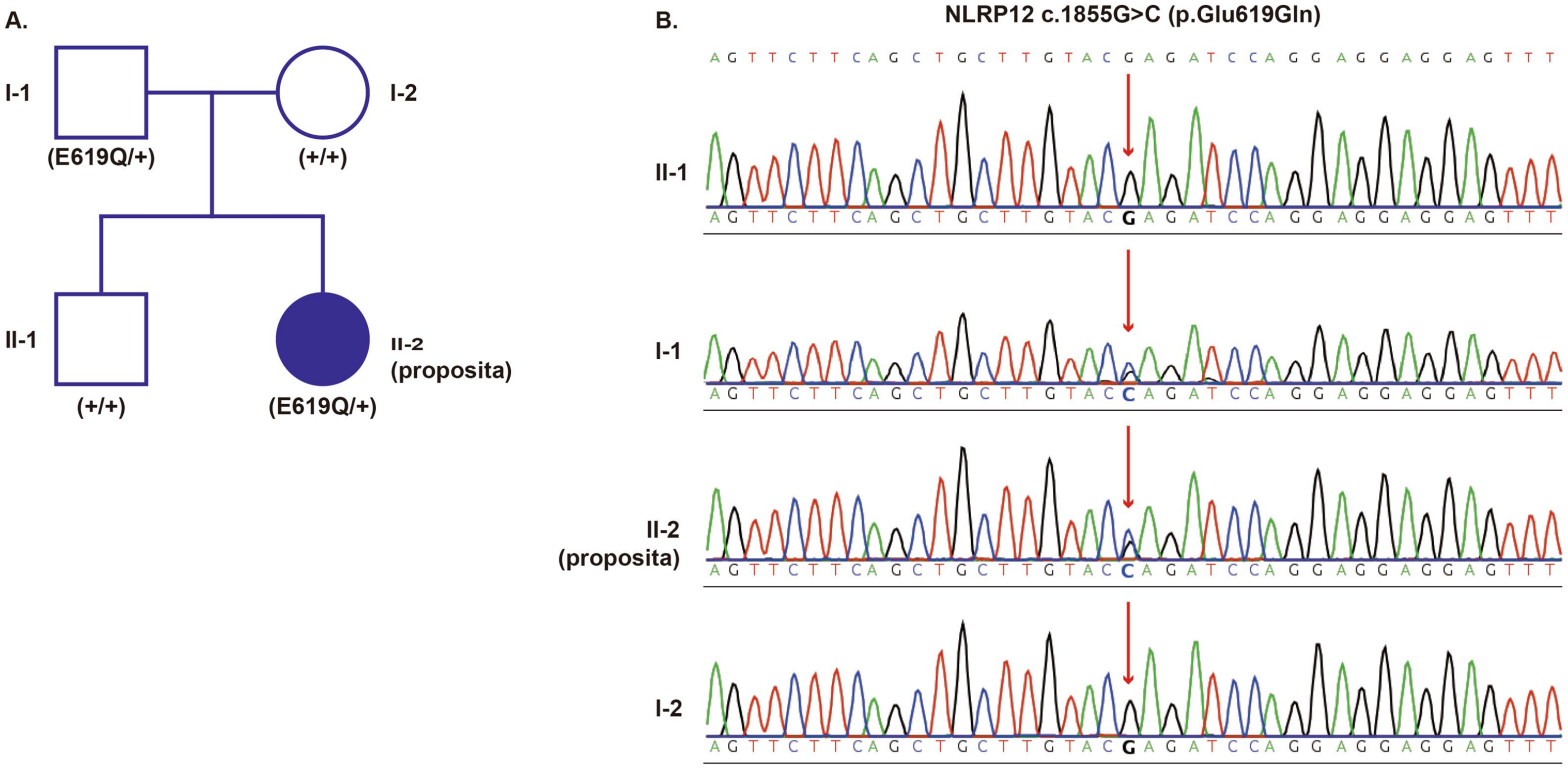
Pedigree, genetic validation, and histopathology. **(A)** Family pedigree showing the proband (I-1, filled symbol) and her asymptomatic father (I-2), who carries the same variant. **(B)** Sanger sequencing confirmed the heterozygous c.1855G > C mutation in the patient and her father.

**Figure 2:**
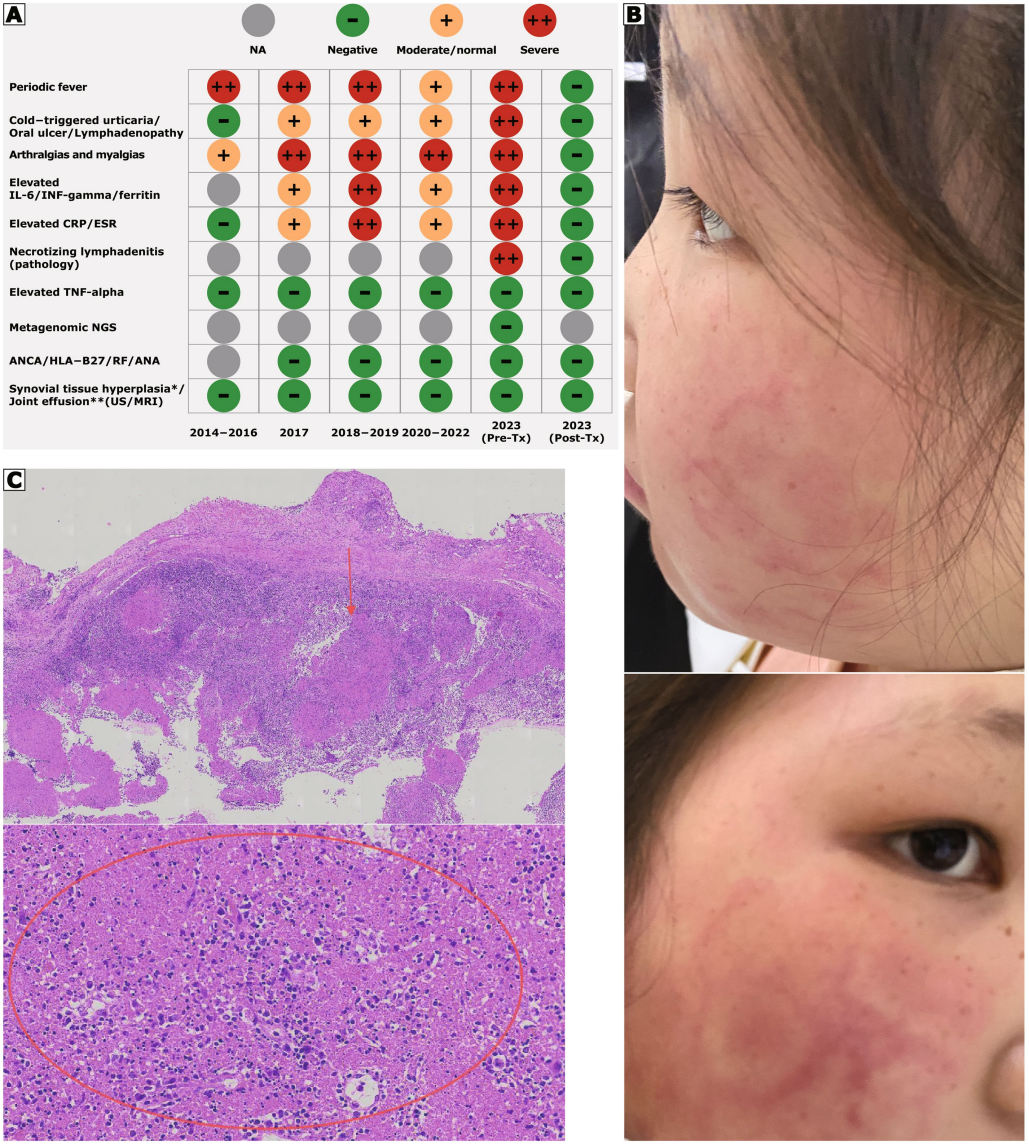
Clinical course and response to treatment. **(A)** Clinical features over time before (Pre-Tx) and after (Post-Tx) starting baricitinib. **(B)** Episodes of cold-triggered urticarial rashes. **(C)** Hematoxylin and eosin (H&E) stained sections of the lymph node biopsy at 10X (lower) and 40X (upper) magnification, showing necrotizing lymphadenitis with karyorrhectic debris and histiocytes. ANCA, antineutrophil cytoplasmic antibody; RF, rheumatoid factor; ANA, antinuclear antibody; HLA-B27, human leukocyte antigen B27; US, ultrasonography; MRI, magnetic resonance imaging. * Bilateral wrists; ** Bilateral knees.

A 16-year-old Chinese girl was referred to our rheumatology center in May 2023 for a 9-year history of recurrent inflammatory episodes, caused by a variant of the NLRP12 gene that was identified (Figure 1). Her symptoms started at age 7, characterized by high-grade fevers (peaking over 39 °C) lasting 3 to 7 days, occurring every 1 to 2 months (Figure 2A). The fevers were consistently accompanied by widespread urticarial rashes triggered by cold exposure (Figure 2B) and polyarticular arthralgia affecting her knees, ankles, and wrists.

### 2.1 Diagnostic journey and previous treatments

As shown in Figure 2A, from 2014 to 2017, her condition was misdiagnosed as recurrent pulmonary infections and treated with multiple courses of antibiotics without success. In 2017, a diagnosis of rheumatic fever was considered due to an elevated anti-streptolysin O (ASO) titer (518.5 IU/mL), despite the absence of clinical signs of infection. Between 2018 and 2019, a diagnosis of juvenile idiopathic arthritis was considered based on external radiological reports suggesting synovitis; however, this was questionable given the negative HLA-B27, rheumatoid factor, and anti-cyclic citrullinated peptide antibodies, and a subsequent review of historical MRI images by our radiologists did not confirm reported sacroiliitis. During this period, treatments included NSAIDs and hydroxychloroquine, which provided only minimal or transient relief and failed to prevent recurrent flares. In contrast, systemic glucocorticoids were effective in aborting acute episodes but were followed by rapid relapse upon discontinuation. Her inflammatory episodes continued.

### 2.2 Diagnostic work-up and key findings

In 2023, her condition worsened, and she developed notable cervical lymphadenopathy. An excisional lymph node biopsy was performed, and histopathological examination revealed necrotizing lymphadenitis (Figure 2C), characterized by karyorrhectic debris and histiocytes with crescent-shaped nuclei, indicative of a systemic inflammatory process. A comprehensive laboratory investigation was conducted. Serum interleukin-6 (IL-6) was persistently elevated at 17.8 pg./mL. In contrast, serum tumor necrosis factor-alpha (TNF-*α*) levels remained within the normal range on multiple measurements. Procalcitonin (PCT) was normal, and peripheral blood microbial cell-free DNA next-generation sequencing (mNGS) failed to identify any pathogenic microbes. These findings collectively argued against an infectious or a typical TNF-mediated autoimmune cause. Tests for antinuclear antibodies, anti-neutrophil cytoplasmic antibodies, and other autoantibodies were negative, effectively excluding systemic autoimmune disorders.

### 2.3 Genetic analysis

**Figure 3:**
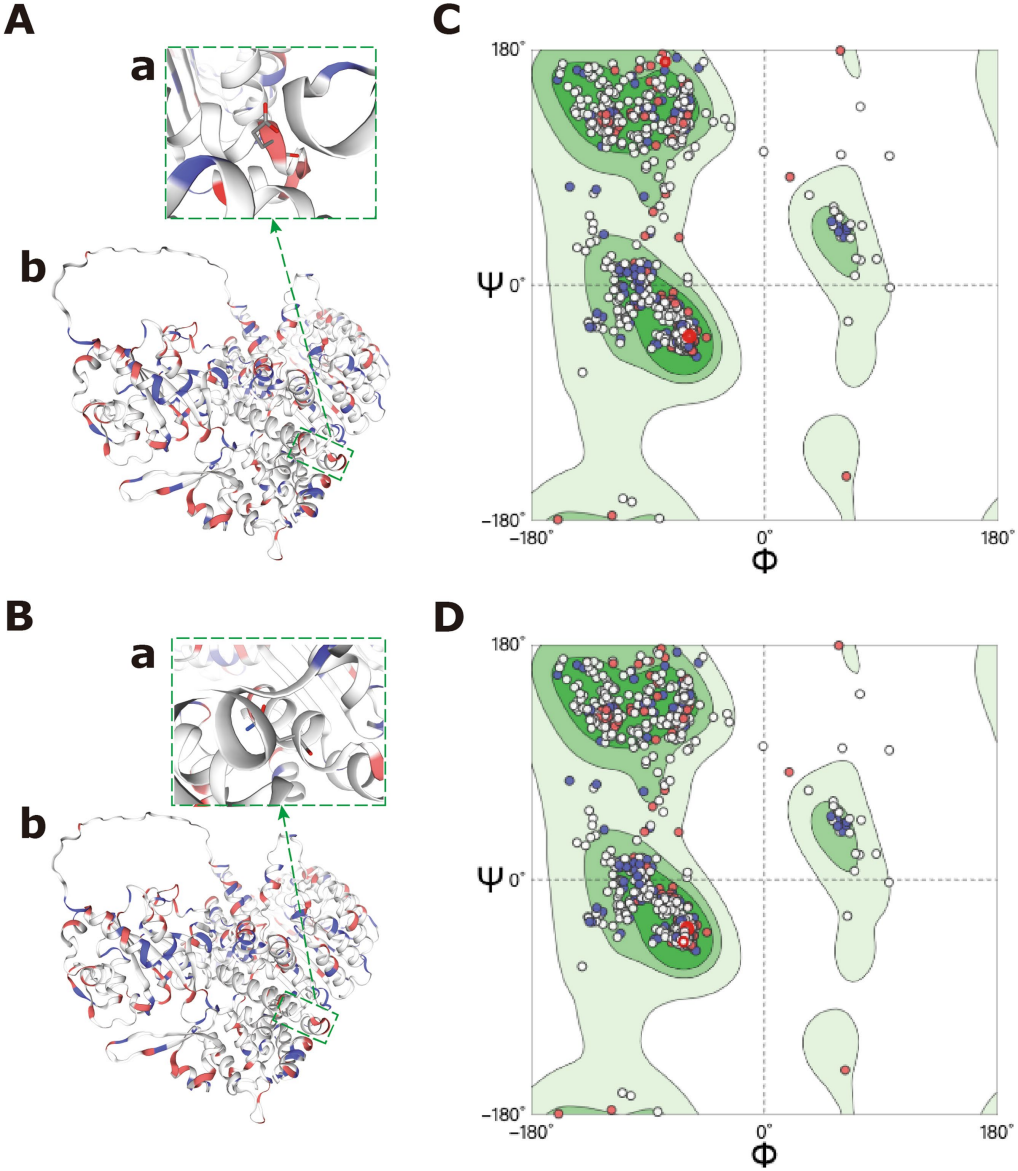
Structural predictions for the *NLRP12* p.Glu619Gln variant. **(A)** Wild-type *NLRP12* structure (HD2 domain in red). **(B)** p.Glu619Gln mutant structure. **(C)** Close-up view of the mutation site, with dashed lines indicating key hydrogen bond interactions disrupted by the substitution. **(D)** Bar graph comparing solvent-accessible surface area (SASA) between wild-type and mutant residues.

Whole-exome sequencing identified a heterozygous *NLRP12* variant (c.1855G > C, p.Glu619Gln). Sanger sequencing validated the variant and showed its presence in the patient’s asymptomatic father, suggesting autosomal dominant inheritance with incomplete penetrance (Figures 1A, B). The variant is very rare (gnomAD allele frequency: 0.00001549) (7, 8) and predicted to be deleterious by several in silico tools (PolyPhen-2: 0.998; SIFT: deleterious) (4). To better understand the potential effect of this HD2-domain variant, we conducted protein structure modeling using AlphaFold2 through SWISS-MODEL (9). Model quality was confirmed with MolProbity (10) and QMEAN (11). AlphaFold2 models indicated that the p.Glu619Gln substitution disrupts critical hydrogen bonds and buries the residue, which could impair *NLRP12*’s autoinhibitory function (Figure 3).

### 2.4 Therapeutic intervention and follow-up

**Figure 4:**
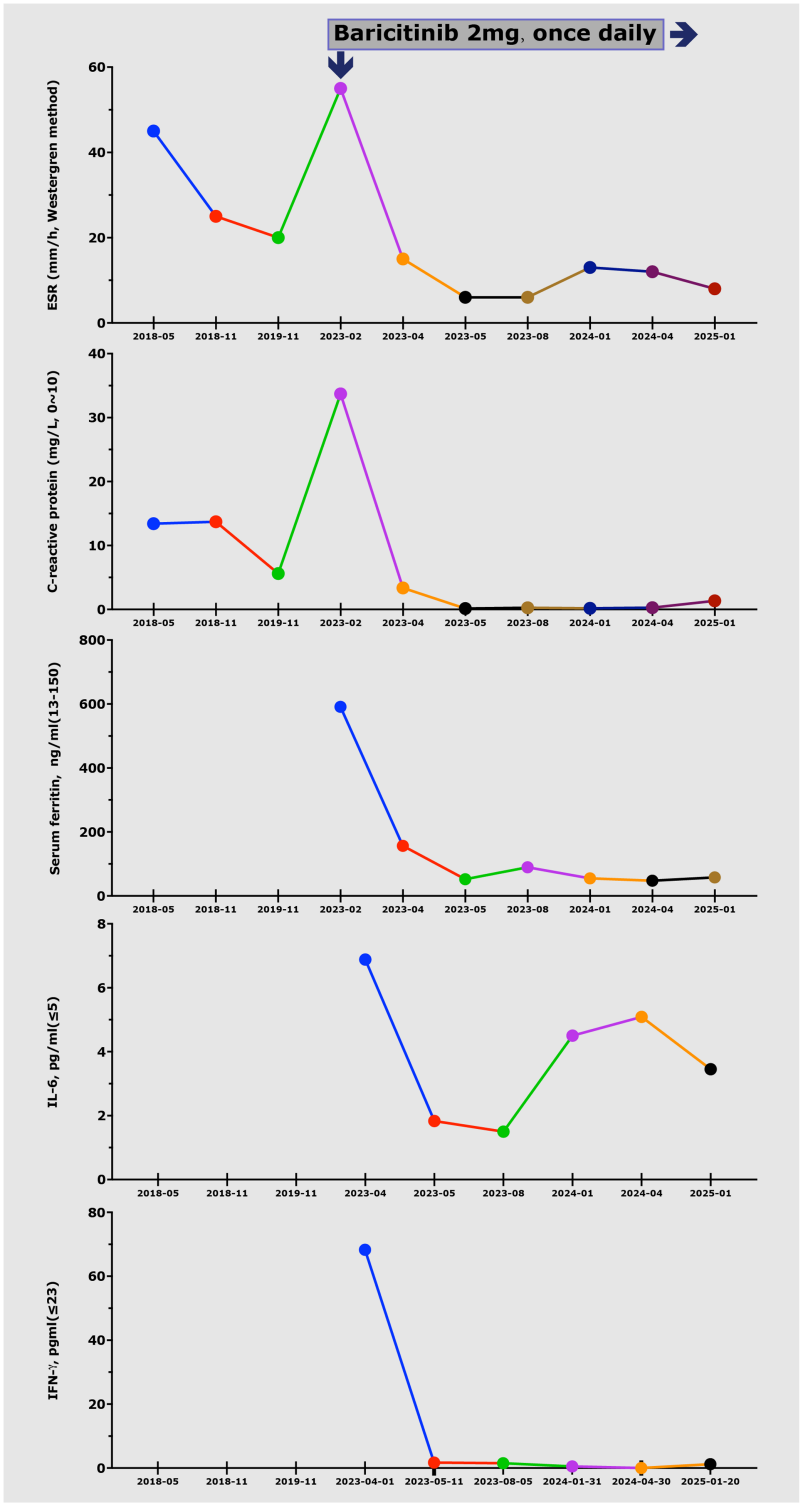
Trend of key laboratory parameters, including IL-6, CRP, serum ferritin, and ESR, demonstrating rapid normalization before and after the initiation of baricitinib treatment.

Based on the elevated IL-6 levels and suspected hyperinflammation, a trial of the JAK inhibitor baricitinib (2 mg/day) was initiated. The patient’s response was rapid and notable. Within two weeks, her fever and rash completely resolved, and her arthralgia significantly improved. Serum IL-6 levels dropped to 2.1 pg./mL (Figure 4). The patient was maintained on 2 mg/day of baricitinib throughout the 12-month follow-up. Routine safety assessments, including complete blood count, liver and kidney function tests, and lipid profile, were conducted quarterly, and no adverse events were observed. She has maintained clinical remission for 12 months and has successfully returned to school.

## 3 Discussion

We present the first clinical case linking the *NLRP12* p.Glu619Gln VUS to a confirmed autoinflammatory disease phenotype. The diagnosis of *NLRP12*-AID in this patient is supported by a substantial body of evidence from clinical, laboratory, histopathological, and therapeutic aspects. The patient’s clinical presentation strongly suggests *NLRP12*-AID, characterized by the classic triad of cold-triggered urticaria, recurrent high fevers, and childhood-onset arthralgia (2). Additionally, we provided objective evidence of systemic autoinflammation, including persistently elevated IL-6 and acute-phase reactants, and most notably, histopathological confirmation of necrotizing lymphadenitis. The normal PCT and negative mNGS effectively excluded an infectious process, while the absence of autoantibodies ruled out typical autoimmune conditions.

Identifying the novel *NLRP12* p.Glu619Gln variant, its extreme rarity in population databases (7, 8), and its classification as a VUS in ClinVar (3), along with computational predictions of its damaging effects, provides a strong genetic basis for the observed phenotype. This finding adds to the expanding genotypic and phenotypic spectrum of *NLRP12*-AID. Notably, as highlighted in a recent comprehensive review, incomplete penetrance is common in *NLPR12*-AID, with about 16% of patients inheriting the variant from an asymptomatic parent (12). This variable expressivity indicates that monogenic predisposition often requires additional genetic, epigenetic, or environmental factors for full clinical expression. Emerging evidence supports this idea: studies have shown that patients with the same *NLRP12* variant can display significantly different clinical features (13), and some children inherit pathogenic variants from completely asymptomatic parents (14). This phenotypic variability may partly be explained by the specific genetic context of the variant itself, such as its location within the gene, which can act as a genetic modifier (15). In our case, the proband’s severe childhood-onset disease might have been triggered by environmental factors like recurrent infections. Meanwhile, the father’s lack of symptoms could result from the absence of such triggers, a protective genetic background, or the potential for the disease to appear later in life. The robust and lasting response to baricitinib offers essential functional confirmation of the diagnosis (16). The patient’s history of poor response to conventional immunosuppressive therapies (NSAIDs, hydroxychloroquine) sharply contrasts with the rapid and sustained efficacy of baricitinib. The quick normalization of IL-6 levels and the resolution of clinical symptoms after JAK inhibition clearly indicate the role of the JAK–STAT signaling pathway in the pathogenesis of this specific *NLRP12* variant. This suggests a mechanism different from the typical IL-1β-driven inflammation often associated with NACHT domain mutations (17) and aligns with emerging evidence of *NLRP12*’s complex role in regulating innate immune signaling beyond the inflammasome (18–20). Although the exact mechanism remains unclear, we hypothesize that the p.Glu619Gln mutation in the HD2 domain may cause JAK–STAT dysregulation through alternative pathways. Since this domain likely influences protein interactions and regulation, the mutation could impair *NLRP12*’s ability to negatively regulate upstream activators of the JAK–STAT pathway. Alternatively, it might enhance *NLRP12*’s role in controlling interferon and cytokine signaling, creating a positive feedback loop that sustains JAK–STAT activation. This genotype–phenotype-therapy connection—a HD2 domain variant linked to high IL-6 levels and intense sensitivity to JAK inhibitors—provides a promising clinical hypothesis for future functional studies.

Although in silico predictions suggested the variant’s harmful potential, this case’s main strength is the detailed clinical and phenotypic evidence. The discovery of incomplete penetrance (asymptomatic father) aligns with the documented variability in *NLRP12*-AID (21).

This case highlights that thorough clinical phenotyping, histopathological correlation, and documenting a targeted treatment response are crucial for determining the pathogenicity of VUS. It not only expands the genotypic spectrum of *NLRP12*-AID but also underscores JAK inhibition with baricitinib as an effective treatment option, especially for HD2-domain variants that may mainly disrupt the JAK–STAT pathway.

## 4 Conclusion

In conclusion, although the presence of asymptomatic carriers within the family demonstrates the incomplete penetrance of *NLRP12*-AID, the converging evidence from genetics, specific phenotyping, histopathology, and a mechanistically consistent therapeutic response provides strong clinical support for the pathogenic role of the *NLRP12* p.Glu619Gln variant. This case underscores the importance of thorough clinical evaluation when interpreting VUS. We recommend reclassifying this variant as “Likely Pathogenic” and suggest that JAK inhibition could be an effective targeted treatment for similar *NLRP12*-AID cases. Final confirmation will depend on functional studies and the identification of additional unrelated cases carrying this variant.

## Data Availability

The original contributions presented in the study are included in the article/supplementary material, further inquiries can be directed to the corresponding author/s.
